# Regulation of T Helper Cell Fate by TCR Signal Strength

**DOI:** 10.3389/fimmu.2020.00624

**Published:** 2020-05-19

**Authors:** Nayan D. Bhattacharyya, Carl G. Feng

**Affiliations:** ^1^Immunology and Host Defense Group, Discipline of Infectious Diseases and Immunology, School of Medical Sciences, Faculty of Medicine and Health, The University of Sydney, Sydney, NSW, Australia; ^2^Tuberculosis Research Program, Centenary Institute, The University of Sydney, Sydney, NSW, Australia

**Keywords:** TCR signal strength, TCR affinity, Antigen dose, CD4^+^ T Cell differentiation, T cell expansion, T cell proliferation, Th1

## Abstract

T cells are critical in orchestrating protective immune responses to cancer and an array of pathogens. The interaction between a peptide MHC (pMHC) complex on antigen presenting cells (APCs) and T cell receptors (TCRs) on T cells initiates T cell activation, division, and clonal expansion in secondary lymphoid organs. T cells must also integrate multiple T cell-intrinsic and extrinsic signals to acquire the effector functions essential for the defense against invading microbes. In the case of T helper cell differentiation, while innate cytokines have been demonstrated to shape effector CD4^+^ T lymphocyte function, the contribution of TCR signaling strength to T helper cell differentiation is less understood. In this review, we summarize the signaling cascades regulated by the strength of TCR stimulation. Various mechanisms in which TCR signal strength controls T helper cell expansion and differentiation are also discussed.

## Introduction

CD4^+^ T helper (Th) cells play a critical role in mediating protective immunity against bacterial, viral, parasitic and fungal infections by regulating the responses of antibody-producing B cells, cytotoxic CD8^+^ T lymphocytes, and macrophages. Naïve CD4^+^ T cells are activated when their unique T cell Receptor (TCR) recognizes cognate peptides presented by Major Histocompatibility Complex (MHC) class II molecules (pMHC-II) in the presence of costimulatory molecules on antigen presenting cells (APCs). To ensure that there are sufficient numbers of antigen-specific clones present to combat pathogens at infection sites, activated CD4^+^ T cells undergo clonal expansion and acquire effector functions in secondary lymphoid organs (SLOs). Depending on the type of pathogen, CD4^+^ T cells can be tailored to become one of several specialized Th subsets defined by their functional attributes (1).

Two prevailing models explain how CD4^+^ T cells integrate different signals to determine lineage commitment. The classic “qualitative” model suggests that Th cell responses are shaped dominantly by the cytokines produced by pathogen-exposed innate cells. The second “quantitative” model proposes that the strength of the signal delivered through the TCR regulates the differentiation program of CD4^+^ T cells. Multiple T cell-associated factors influence the overall strength of TCR signaling strength. These include the quality of the interactions between MHC and TCR molecules, the amount of antigen, and the degree of costimulation (2). This review will focus mainly on how the potency of signals delivered through the TCR controls the activation, expansion, and differentiation of CD4^+^ T cells.

## Experimental Approaches Used to Manipulate TCR Signal Strength

Different experimental approaches have been used to study the role of TCR signal strength in controlling T cell responses. One of the most frequently employed methods is to titrate the amount of antigen thereby altering the number of peptide-occupied MHC molecules that engage cognate TCRs (2). Another method is to induce mutations in peptide sequences (Altered Peptide Ligands, APLs), which allows investigators to change the potency of TCR signals by altering antigen-binding affinity to MHC molecules and TCR complexes (2, 3). In addition, by altering pathogen or adjuvant doses, variations in the degree of inflammation modulate the intensity of costimulatory signals delivered through CD28, and hence the overall strength of TCR signals (4). It is important to note that because the TCR itself is not modified, the approaches mentioned above cannot analyze the contribution of T cell-intrinsic variations in TCR signal strength to the T cell response.

One T cell-intrinsic factor that affects the strength of the signal delivered through the TCR is the number of TCR molecules expressed, also known as TCR avidity (5). Manipulation of TCR avidity achieves similar outcomes as changes in antigen dose or increasing the number of peptide-loaded MHC molecules, and is naturally achieved after initial T cell activation in a process known as avidity maturation (6). Alternatively, by introducing mutations into TCR sequences, investigators can change the affinity of the TCR for pMHC complexes (7). This alters the binding kinetics between TCR and pMHC complexes such that high affinity TCRs bind with slower off/dissociation rates (K_d_) when measured using two-dimensional (2D) kinetic assays. Hence at the level of an individual TCR, slower K_d_'s lead to prolonged pMHC:TCR interactions (2). Alterations in antigen dose and the degree of costimulatory signaling also affect the duration of contact between APCs and T cells (8, 9). Higher antigen levels result in longer interaction or “dwell” times and confer potent, prolonged TCR signals; whereas lower antigen dose stimulation results in shorter interactions and impart protracted, weaker signals (9). However, higher-affinity TCRs do not always facilitate greater dwell times or signal strength (10, 11). It is therefore unclear whether higher affinity TCRs accelerate T cell activation by decreasing the antigen threshold required for activation (12–14), or whether they promote longer interactions to stabilize lineage commitment following T cell activation (9).

## TCR Signaling Cascade

The TCR complex consists of a variable heterodimer (TCRαβ) that binds to antigenic ligands and the invariant signaling component, CD3, which is composed of gamma, delta, epsilon, and zeta subunits. Whilst there is much debate on how TCR signals are initially triggered [reviewed by (15)], it is generally agreed that TCR ligation to agonistic pMHCs results in the aggregation of TCR-CD3 complexes with costimulatory and adhesion proteins such that an immunological synapse is formed (16, 17). The physical force of pMHC:TCR binding is thought to change the orientation of cytosolic signaling components of the TCR-CD3 complex (18, 19). This change results in the phosphorylation of Immunoreceptor Tyrosine-based Activation Motifs (ITAMs) by Src family Protein Tyrosine Kinase (PTK), such as Lymphocyte-specific protein tyrosine Kinase (LCK) (20, 21). The concurrent exclusion of constitutively active transmembrane tyrosine phosphatases, such as CD45 (that oppose the activity of constitutively active PTKs) away from phosphorylated ITAMs ensures that TCR signals are maintained in a pMHC:TCR-dependent manner (22–24).

**Figure 1:**
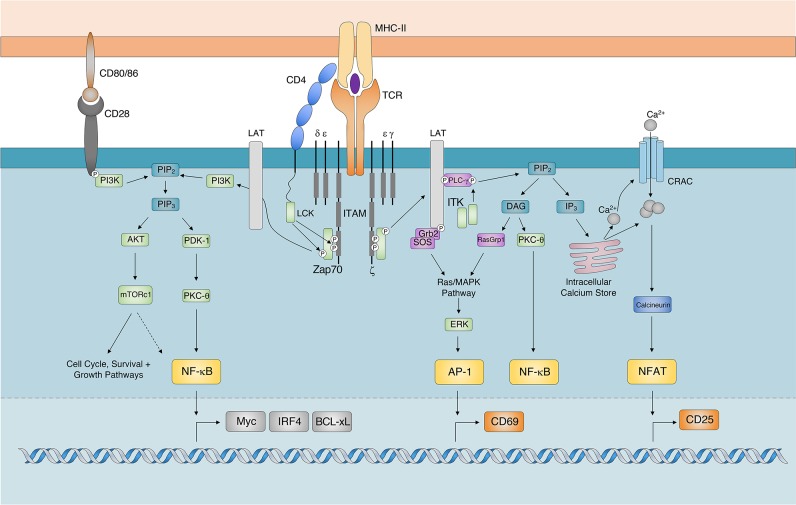
TCR Signaling Cascade. The major signaling components and transcription factors responsible for the transduction of TCR signals following the recognition of cognate pMHC-II. While AKT dependent activation of mTOR signaling regulates cell proliferation, survival, and growth pathways it has also been shown to regulate the expression of Myc, IRF4, and BCL-xL by potentially regulating (indicated by dashed arrow) the activity of NF-κB. AP-1, Activator Protein 1; AKT, Protein Kinase B; BCL-xL, B Cell Lymphoma-extra Large; DAG, Diacylglycerol; CRAC, Calcium Release Activated Channel; ERK, Extracellular signal-Regulated Kinase; GRB2, Growth factor Receptor-Bound protein 2; IP_3_, Inositol-1,4,5-trisphosphate; ITAM, Immunoreceptor Tyrosine-based Activation Motif; IRF4, Interferon Regulatory Factor 4; ITK, Interleukin-2-inducible T cell kinase; mTORc1. Mechanistic Target Of Rapamycin complex 1; NFAT, Nuclear Factor of Activated T cells; NF-κB, Nuclear Factor kappa-light-chain-enhancer of activated B cells; PLC-γ, Phospholipase C-gamma; PDK-1, Phosphoinositide dependent kinase-1; PKC-θ, Protein Kinase C theta; PIP_2_, Phosphatidylinositol-4,5-bisphosphate; RASgrp1, RAS guanyl-releasing protein 1; TCR, T cell Receptor; SOS, Son of Sevenless; Zap70 ζ-chain associated protein kinase of 70 kDa).

Ligation of the TCR, also facilitates the recruitment and docking of the CD4 co-receptor to the pMHC-II complex (25). This positions CD4-associated LCK in locations favorable for ITAM phosphorylation on cytosolic TCR/CD3 complexes (Figure 1). Activated ITAMs serve as docking sites for the Src Homology 2 (SH2) domains of Zeta chain of T cell receptor Associated Protein kinase 70 (Zap70) (26, 27). Importantly, as TCR-CD3 complexes continue to aggregate into microclusters (15), Zap70 undergoes a conformational change associated with enhanced binding affinity and recruitment to ITAMs (28, 29). Zap70 is subsequently activated following its phosphorylation by LCK. Given continued pMHC:TCR-CD3 interactions and receptor clustering, activated Zap70 is released from ITAMs (30) and subsequently phosphorylates signaling scaffold adaptor proteins, such as Linker for Activated T cells (LAT) and SH2 domain containing Leukocyte Protein of 76 kDa (SLP-76) (31). Activation of these adaptor proteins provide SH2- and SH3-binding sites for the initiation of Phosphatidylinositol 3-Kinases (PI3K), Protein Kinase B (AKT) (32), Inducible T cell Kinase (ITK) and Protein Kinase C (PKC) dependent signaling cascades (16, 33). PI3K signaling results in the Phosphoinositide-dependent protein kinase-1 (PDK-1) dependent activation of PKC-θ for the activation and nuclear translocation of Nuclear Factor Kappa-light-chain-enhancer of activated B cells (NF-κB) (34–36).

PI3K activity also results in the AKT-dependent activation of Mechanistic Target Of Rapamycin (mTOR). Additionally, activated ITK phosphorylates the lipase Phospholipase C gamma (PLCγ), which ultimately cleaves Phosphatidylinositol-4,5-bisphosphate (PIP_2_) in the plasma membrane to generate the secondary messengers inositol-1,4,5-triphosphate (IP_3_) and Diacylglycerol (DAG) (37). The accumulation of ITK promotes the downstream release of calcium ions (Ca^2+^) from endoplasmic reticulum (ER) Ca^2+^ stores (38). The release of intracellular calcium, through the effects of calcineurin, activates and promotes the nuclear translocation of Nuclear Factor of Activated T cells (NFAT) (39). The ITK-dependent generation of DAG results in the recruitment of Ras Guanyl-Releasing Protein 1 (GRP1) and PKCθ for the activation of the Mitogen Activated Protein Kinase (MAPK)/Extracellular signal Regulated Kinase (ERK) and NF-κB pathways, respectively (40). Activation of the MAPK/ERK signaling cascade triggers the formation of the transcription factor Adaptor-related Protein complex 1 (AP-1) (41). Together, the nuclear translocation and transcriptional activity of AP-1, NFAT and NF-κB orchestrate T cell activation.

## TCR Signal Strength Regulates the Activity of TCR Signaling Components

The strength of the input delivered through the TCR is translated into digital/“all or nothing,” or analog/“scaled” alterations to the TCR signaling cascade (42, 43). In this way potent TCR signals regulate the activity of important transcription factors critical in determining the fate of T cells.

### Potent TCR Signals Scale the Activity of Zap70 and PI3K

Strong TCR signals delivered through pMHC:TCR interactions are amplified in accordance to the duration of time ITAMs remain phosphorylated for the subsequent recruitment and activation of Zap70 (9). When compared to lower affinity counterparts, high affinity TCRs bind cognate pMHCs with distinct docking geometries (44) that ultimately result in the clustering of receptors (29), as well as a mechanical “push” or “pull” of the TCR (19). Whilst the exact mechanism is unclear, the magnitude of this interaction is dependent on TCR/antigen affinity and dictates the type of bond formed between T cells and APCs (45). While low affinity TCRs confer weaker pulling forces that result in the formation of slip-bonds, high TCR affinity induces the formation of catch-bonds (19, 46). Catch-bonds extend the duration of pMHC:TCR interactions and promote the exclusion of CD45 and its intracellular inhibitory phosphates from the immunological synapse (10, 19, 47). Additionally, high affinity pMHC:TCR binding events have recently been shown to enhance the recruitment and retention of CD4 co-receptors in the pMHC:TCR cluster (48) such that CD4-assoicated LCK can further sustain ITAM activation on cytosolic TCR/CD3 chains. Hence, by tipping the balance of activating PTKs to inhibitory phosphatases in close proximity to TCR/CD3 complexes in favor of activating PTKs, strong TCR signals extend the duration in which ITAMs on TCR/CD3 chains remain phosphorylated. As such, longer pMHC:TCR interactions have been shown to enhance the recruitment and activation of Zap70 in bulk (49–51) and single (29, 52) cell analyses.

In addition, strong TCR signals enhance the activity of phosphorylated Zap70 (28). Importantly, when low potency TCR signals are delivered, in addition to phosphorylating ITAMs, LCK phosphorylates Src Homology region 2 domain-containing Phosphatase-1 (SHP-1). During low potency TCR stimulation, phosphorylated SHP-1 inactivates the kinase activity of LCK (53) and Zap70 (54) which results in the attenuation of TCR signals. In contrast to this, strong TCR signals result in a greater proportion of cells expressing phosphorylated ERK which itself phosphorylates LCK and prevents the binding of SHP-1 (53). These results suggest that strong TCR signals resulting from longer pMHC:TCR interactions augment the quantity, quality and duration of Zap70s catalytic action, extending the duration in which downstream signaling cascades remain active.

Through its ability to scale the activity of Zap70, sustained interactions between pMHC:TCR complexes promote the recruitment of PI3K to the adaptor protein LAT (32, 55). Activated PI3K phosphorylates PIP_2_ for the generation of the secondary messenger Phosphatidylinositol (3,4,5) triphosphate (PIP_3_). Scaled increases in PIP_3_ concentrations regulate both the recruitment of AKT and the activation of its serine/threonine kinase activity (56). AKT indirectly activates mTOR, whose kinase activity is critical for preparing activated T cells for the bioenergetic demands of clonal expansion. Whilst it is well-established that TCR signaling can activate mTOR in a PI3K/AKT-dependent manner (57), TCR-dependent activation of mTOR is considered weak and transient (58). Nevertheless, initial mTOR expression is scaled in an antigen dose-dependent manner (58, 59), suggesting that whilst transient, TCR signals can directly scale the expression of mTOR. In addition, CD28 ligation leads to the phosphorylation of its cytoplasmic tail and the subsequent recruitment of PI3K (60, 61) for the activation of AKT (62) and mTOR (57, 63). As such, PI3K/AKT can be posited as a rheostat that modulates the amplitude of mTOR expression in accordance to the strength of signals delivered through costimulatory molecules.

### Dynamic Regulation of NF-κB, NFAT, and ERK by TCR Signal Strength

The activation and nuclear translocation of NF-κB in CD4^+^ T lymphocytes regulates cell survival by controlling the expression of Interleukin (IL) 2 (IL-2) and anti-apoptotic proteins (64–67). TCR/ITK and CD28/PI3K signaling pathways can independently activate PKC-θ for the downstream activation and nuclear translocation of NF-κB (35) (Figure 1). Once a threshold TCR signal has been achieved, TCR and CD28 signaling results in the digital activation and nuclear translocation of NF-κB (68). Despite this, TCR signal strength has been shown to scale the expression of NF-κB-dependent targets, such as pro-survival molecule B-Cell Lymphoma-extra large (BCL-xL) (69) and the transcription factor IRF4 (70, 71). This suggests that whilst TCR signal strength does not regulate the amplitude of NF-κB expression, it may control its transcriptional activity. Alternatively, there is evidence to suggest that the TCR signal strength-sensitive PI3K/AKT/mTOR pathway can modulate NF-κB activity (Figure 1). AKT is proposed to regulate the duration of NF-κB nuclear translocation (72, 73), as well as the range of genes it targets for transcription (74). Albeit in a cancer cell line, the ability of AKT to control NF-κB activation was shown to be reliant on mTOR (75). As mTOR is known to regulate the antigen affinity-driven expression of IRF4 in CD8^+^ T cells (76), these findings further suggest that the PI3K/AKT/mTOR signaling cascade may translate analog TCR inputs into analog NK-κB transcriptional outputs.

Strong TCR signals have historically been shown to amplify the accumulation of intracellular calcium (14, 77, 78). It is believed that strong TCR signals increase the number activated ITKs recruited to facilitate the IP_3_-dependent accumulation of intracellular Ca^2+^ (38, 79, 80). The magnitude of Ca^2+^ release into the cytosol has traditionally been associated with increased levels of activated, nuclear NFATc (81, 82). Despite this, single cell analyses have revealed analog increases in intercellular calcium translate into digital, “all or nothing” NFATc1 or NFATc2 expression patterns in the nuclei of CD4^+^ T cells (83–88).

**Figure 2:**
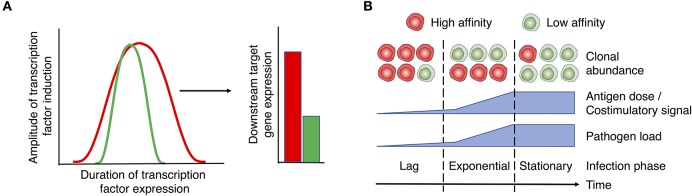
Proposed role of TCR signal strength in T cell function and expansion. **(A)** TCR signals initiate the “all or nothing” digital induction of transcription components, such as ERK, NF-κB, and NFAT. The amplitude of their expression is independent of the strength of input signals. Instead, TCR signal strength determines the duration of transcription factor activity (left panel), translating the digital expression pattern of transcription factors into the analog expression of some downstream targets like CD69, CD25, Myc, and IRF4 (right panel). Red and green lines/bars denote strong and weak TCR signals and the associated responses. **(B)** T cell activation and expansion are regulated by the interplay between T cell-intrinsic factors (e.g., TCR affinity) and extrinsic factors (e.g., the levels of antigen and costimulation associated with different disease stages). In the case of a persistent infection, the relative abundance of clones with high vs. low affinity TCRs may vary depending on the stage of infection. Early stages of infection, when low levels of antigen/costimulation are present, favor the expansion of clones with high affinity TCRs. As pathogen levels rise, lower affinity clones are able to undergo activation and equivalent expansion. As a potential strategy to prevent immunopathology during persistent stages of infection, clones with high affinity TCRs may be removed or silenced from the effector T cell pool. Therefore, low affinity clones may become the major population of effector T cells during chronic infection. Red and green cells denote T cells with high and low affinity TCRs, respectively.

Similarly, the MAPK/ERK signaling cascade exhibits a digital signal response and hence is not scaled by TCR signaling (42, 89, 90). The digital expression of NFAT and ERK in response to analog TCR/ITK input is thought to be a result of positive feedback circuits that reinforce NFAT (91, 92) and ERK (89) expression once a threshold of activation has been reached. NFAT and ERK/AP-1 predominantly drive the expression of surface markers associated with CD4^+^ T cell activation, such as CD25 (93, 94) and CD69, respectively (89, 95). Importantly, recent single cell analyses indicates that TCR signal strength scales their expression (84, 85, 96). How then does the digital expression of NFAT/ERK translate into analog downstream gene/protein expression? In the case of NFAT, the extent of initial pMHC:TCR interactions has been shown to modulate the duration of its activity in the nucleus, even after TCR signals have ceased (97). Therefore, similar to the regulation of NF-κB activity discussed above, whilst TCR signal strength may not directly control the magnitude of NFAT or ERK expression, it may extend the duration in which these transcription factors remain active, which subsequently leads to greater expression of target genes and their functional outputs (Figure 2A). Future studies need to delineate how TCR signal strength influences the magnitude or duration of downstream signals to achieve functional differences.

## The Potency of TCR Signals Dictate the Magnitude of T Cell Expansion

Control of microbial infection requires the rapid generation of large numbers of effector Th cells. While naïve CD4^+^ T cells acquire the ability to produce IL-2 following activation by a single agonistic pMHC-II complex (52, 98), enduring Th cell proliferation appears to require stronger TCR stimulation (99) with an estimated 50–400 pMHC-IIs being required (100, 101). The ability for strong TCR signals to promote the expansion of cytotoxic and Th cells is well-established (102). For example, CD8^+^ T cells primed by high affinity antigens *in vivo* underwent greater expansion when compared to those stimulated by low affinity antigens (103–105). Moreover, CD4^+^ T lymphocytes with high affinity/avidity TCRs (106–113) or those that were stimulated with high affinity antigens or doses (71, 114–118) generally underwent greater expansion *in vivo*. In general, TCR signaling can scale the magnitude of T cell expansion by coordinating the tightly linked metabolic and cell cycle pathways. These programs determine the extent of T cell expansion by regulating the time taken for individual cells to enter and then progress through the cell cycle, the rate of subsequent proliferation and the cells proliferative capacity (119).

Stimulation with high concentrations of antigen or enhanced cross-linking of CD3 accelerates the progression of naïve Th cells from senescence (G_0_) into the Gap phase 1 (G_1_) of the cell cycle (120), reducing the time it takes for CD4^+^ T cells to start dividing *in vitro* (121, 122). Whilst not studied in CD4^+^ T cells, high antigen affinity and doses have been shown to reduce the time to first division in CD8^+^ T cells (123). In addition to reducing the time taken for Th cells to undergo their first division (124), potent CD28 ligation enhances the subsequent rate of proliferation in CD4^+^ (120) and CD8^+^ T cells (125) *in vitro*. Although IL-2 signaling is proposed to regulate the subsequent rate of cell division in CD8^+^ T cells, the contribution of IL-2 to the rate of Th cell proliferation is unclear, with negligible (126) and supportive (121) roles being reported. Hence, the mechanism by which strong TCR signals accelerate Th cell progression through the first and subsequent cell cycles remains incompletely understood.

One potential mechanism by which TCR signal strength controls Th cell proliferation is by scaling the activity of mTOR. It is well-established that progression from G_0_ is the result of the PI3K/mTOR complex 1 (mTORc1) dependent expression of cyclins and cyclin dependent kinases (CDKs) (127–130). Additionally, mTORc1-dependent upregulation of the Large neutral Amino acid Transporter (LAT1, CD98) and Glucose Transporter 1 (GLUT-1) facilitates nutrient uptake and sustains T cell growth during the G_1_ phase (57). Interestingly, although reduced, continuous T cell proliferation can occur when mTORc1 signaling is low or absent (63, 130–132). As activated T cells continuously proliferate by entering the cycle from the G_1_ phase (133), these findings suggest that a major function of mTOR may be to initiate cell cycle entry and regulate the timing of initial T cell division in a TCR signal strength-dependent manner.

The number of times a T cell divides before it senesces is also known as the proliferative capacity or Division Destiny (DD) (125). Potent CD28 stimulation, antigen affinity or antigen doses have separately been shown to enhance the proliferative capacity of CD8^+^ T cells (123, 125). Recent findings have suggested that the amplitude of Myc expression prior to a T cell entering their first division enables them to undergo a greater number of divisions (134, 135). Given the short half-life of Myc, the greater the nuclear concentration of Myc, the longer it takes for Myc levels to fall below a threshold that can promote cell division. In this way, Myc acts as a timer for the DD of a cell. Interestingly, changes in the strength of CD28 signaling, but not antigen affinity or dose, scale the amplitude of Myc expression in CD8^+^ T cells (134, 135). As both PI3K and NF-κB have both been shown to regulate the expression of Myc (136, 137), these data may suggest that TCR and CD28 signals differentially scale the expression of cell division related transcription programs. Supporting this are observations that increased antigen affinity and dose enhance the amplitude and the transcriptional specificity of IRF4 in CD4^+^ T cells (71, 138), whereas increases in CD28 stimulation have a minor impact on IRF4 expression (139). The amplitude of IRF4 expression is known to control the magnitude and duration of CD8^+^ T cell expansion by regulating aerobic glycolysis (140), inhibiting repressors of the cell cycle and by preventing the expression of the pro-apoptotic protein Bim (76). As TCR signals also promote IRF4 expression in an mTOR- and NF-κB-dependent manner (70, 76), these data suggest that whilst the TCR and CD28 signal through overlapping pathways (i.e., PI3K, NF-κB), they differentially regulate the cumulative strength of this signal and the resulting proliferative capacity of T cells, possibly by controlling distinct sets of transcription factors (such as Myc and IRF4).

The presence of Th cells with high affinity TCRs may be critical in mobilizing T cell responses to invading pathogens during early stages of infection when pMHC-II and tissue inflammation levels are low. As Th cells with high affinity TCRs possess a lower antigen activation threshold, they may enter the cell cycle quicker than their low affinity counterparts, such that they are preferentially recruited into the activated T cell pool. This accelerated response provides an early burst of effectors that may be critical for preventing the dissemination of infection (Figure 2B). However, if antigen levels rise or persist, as is the case with persistent infections, T cell clones with lower affinity TCRs may come to dominate the effector cell pool. Given greater antigenic and inflammatory signals, high affinity effectors may succumb to Activation-Induced Cell Death (AICD) or exhaustion (141–143). Moreover, high affinity effectors may be functionally silenced from the immune response to prevent potential immunopathology through the downregulation of the TCR (144). Therefore, during the persistent phase of an infection, lower affinity clones are less likely to be out-competed by their high affinity counterparts (145, 146) and are able to undergo activation and expansion (Figure 2B). This dynamic may account for recent observations that lower affinity/avidity clones make up a large and underappreciated fraction of responding Th cell populations during late stages of infection (144, 146–152).

Given that the overall strength of the TCR signal is a sum of TCR affinity, pMHC-II densities and costimulatory signals, which are in part dictated by the intensity of infection, TCR signaling can be highly dynamic as pathogen loads vary during different stages of infection. This allows T cells with a spectrum of affinities to be effectively stimulated and sufficient numbers of effector T cells to be generated to contain invading pathogens regardless of the stage of infection. Taken together, it is likely that TCR affinity, antigen potency, and antigen dose play dynamic and potentially distinct distinct roles in the regulation of Th cell expansion. This has been proposed by other studies, albeit in models lacking inflammation (153, 154). Future studies will need to formally investigate these hypotheses by dissecting the interplay between changes in TCR affinity, the timing of clonal contraction and by examining how changes in pathogen levels affect this dynamic across different tissues.

## TCR Signaling Strength and CD4^+^ T Cell Differentiation

The role of TCR signal strength in CD4^+^ T cell differentiation has traditionally been determined by investigating the ability of naïve Th cells to commit to one effector lineage over the other in response to stimulation with different model antigens. Early *in vitro* investigations into the role of TCR signal strength have generally reported that stimulation with high antigen doses favored IFN-γ over IL-4 production in TCR transgenic T cell cultures (155–160). However, high dose antigen stimulation has also been shown to promote IL-4 production *in vitro* (161–165). This discrepancy might be explained by variations in mouse strains used between studies. For example, TCR transgenic cells with a B10.A genetic background favored IFN-γ production (155, 159), whereas those on a BALB/c background skewed to IL-4 production (161, 163). Intriguingly, the same TCR transgenic T cells from the BALB/c mice that predominantly produced IL-4 in response to high dose antigen stimulation (161, 163–165) have been shown to favor IFN-γ production in other studies (156, 157), suggesting that the outcome of T cell differentiation is influenced by *in vitro* culture conditions.

Since strong TCR signals as a result of high antigen dose stimulation promote IL-4 over IFN-γ expression under some circumstances (161, 163–165), a bi-phasic Th2 differentiation model has been proposed by Nakayama and Yamashita (166). This model hypothesizes that naïve CD4^+^ T cells can differentiate into IL-4-expressing cells in the presence of both very low- and high-levels of cognate antigen. Although activation of ERK by strong TCR signals reduces IL-4 expression in peptide-activated Th cells (159, 167), ERK signaling has been shown to promote IL-4 expression in CD4^+^ T cells stimulated with TCR cross-linking antibodies (166). These findings suggest that ERK could play a dual role in TCR signal strength-dependent Th2 differentiation.

On the other hand, strong TCR signaling as a result of stimulation with high affinity APLs has generally favored the generation of IFN-γ over IL-4 producing effectors *in vitro* (81, 158, 167–170) and *in vivo* (171). Furthermore, when two TCR transgenic T cell lines recognizing the same antigen were compared *in vitro*, CD4^+^ T cells with weak TCR signaling due to a mutation in the TCR alpha chain were shown to favor IL-4 induction (172). Studies that have attempted to uncover the mechanism that results in enhanced Th1 differentiation *in vitro* have suggested that strong TCR signals prevent default Th2 programs rather than actively driving Th1 polarization. Here, strong TCR signals are believed to prevent the early expression of IL-4, and its autocrine signaling that results in the expression of GATA Binding Protein 3 (GATA3) for Th2 differentiation (1, 159), by enhancing the nuclear translocation of NFATp (81) and altering the DNA binding activity of AP-1 (167). Some studies have demonstrated that potent costimulation or stimulation with high antigen doses can actively promote the generation of IFN-γ-expressing effectors by regulating the ability of CD4^+^ T cells to respond to the Th1 polarizing cytokine IL-12 (4, 173). IL-12 is known to promote Th1 differentiation and IFN-γ expression by promoting the expression of T-box binding transcription factor (T-bet), the master regulator of Th1 polarization (1). While these studies associated high antigen dose stimulation with enhanced IL-12 Receptor Beta 2 (IL-12Rβ2) chain expression, the mechanism linking the two events remains to be identified.

There is also evidence to suggest that strong TCR signaling may indirectly promote Th1 differentiation by altering the function of APCs. Here, stimulation of CD4^+^ T cells with high affinity antigens has been shown to enhance the production of IL-12 from co-cultured APCs by enhancing the expression of CD40L on developing Th cells *in vitro* (157, 174, 175). In summary, when comparing Th1 and Th2 differentiation *in vitro*, the majority of studies indicate that strong TCR signals seem to favor the generation of Th1 effectors by directly preventing the early expression of IL-4 and GATA3, as well as by promoting the expression of the IL-12Rβ2. Importantly, T-bet is known to drive IFN-γ expression and prevent Th2 differentiation by sequestering GATA3 (176–178). Hence it is likely that strong TCR signals further favor the generation of Th1 over Th2 cells by promoting IL-12-dependent T-bet expression for the subsequent sequestration of GATA3. Future studies will need to formally investigate how potent TCR signals regulate the crosstalk between T-bet and GATA3 for the generation of Th1 and Th2 effectors, respectively.

Interestingly, early *in vivo* studies have demonstrated that high antigen dose stimulation promotes the generation of IL-4 producing effectors (179–182). Whilst this appears to support the *in vitro* findings underlying the signal strength model proposed by Nakayama and Yamashita (166), the apparent Th2 phenotype was also accompanied with greater levels of class-switched antibodies, which are now recognized to be a result of IL-4 producing follicular T helper cells (Tfh) that are difficult to stably generate *in vitro* (183–185). Hence these studies provided the initial indication that high antigen doses promoted the generation of Tfh cells *in vivo* and may help build on findings that indicate that medium-strong TCR signaling can promote the generation of IL-4 expressing effectors *in vitro*. This has since been confirmed in recent *in vivo* studies where high antigen dose stimulation resulted in longer dwell times, and favored the generation of Tfh cells over Th1 effectors (11, 115, 186–188). As IL-2 signaling is known to suppress the expression of BCL-6, the master regulator of Tfh differentiation (189), it was proposed that high antigen dose stimulation favored Tfh cell generation by reducing IL-2 signaling (188).

Intriguingly, when the responses of two TCR transgenic populations that recognize the same antigen with different binding affinities were recently compared *in vivo*, a greater proportion of high affinity TCR-bearing Th cells were found to undergo Th1 commitment (108). Moreover, stimulation with high affinity antigens have also been shown to favor the generation of Th1 effectors (115, 190). It is thought that high affinity TCR interactions drive STAT5-dependent Th1 differentiation (191) by upregulating and maintaining the expression of the high affinity IL-2 receptor, CD25 (108, 192). Although high affinity TCR interactions generally favor Th1 differentiation, high affinity TCRs have been shown to promote the generation of Tfh cells (107) or play a more capricious role (11). As has been suggested previously (115), these data again suggest that antigen dose and TCR affinity play distinct roles in determining the fate of Th cells. Therefore, while high antigen dose stimulation favors the generation of Tfh cells *in vivo*; the function of TCR affinity in determining Th differentiation may be secondary to T cell-extrinsic factors such as innate cytokines. Hence, the role of TCR affinity in lineage differentiation may vary depending on the experimental model employed.

The strength of TCR signaling has also been suggested to regulate Th17 differentiation. Under Th17-polarizing conditions *in vitro*, high antigen dose and persistent TCR stimulation has been shown to promote IL-17 expression (193, 194). Furthermore, when TCR signals are attenuated through the deletion of ITK, CD4^+^ T lymphocytes were more prone to differentiate into Forkhead box P3 (Foxp3) expressing regulatory Th cells (Tregs) over IL-17 producing cells (195). However, low levels of CD28 costimulation or antigen stimulation, as well as exposure to low potency antigens, have each been shown to favor the generation of IL-17 over IFN-γ (196, 197) or IL-4 expressing effector CD4^+^ T cells (198). Therefore, the role of TCR signal strength in Th17 differentiation remains unclear.

Distinct mechanisms for how TCR signal strength modulates Th17 polarization were proposed in these studies. TCR dependent AKT signaling was shown to be associated with both enhanced (195) and reduced (197) Th17 differentiation. Moreover, antigen dose dependent scaling of CD40L expression on activated Th cells was shown to promote IL-6 production by APCs for the enhanced generation of Th17 cells *in vitro* (193). Regardless, the generation of IL-17 expressing effectors was shown to be dependent on exposure to the correct cytokine milieu (193–198). This suggests that when compared to polarizing cytokines, TCR signal strength may play a secondary, context-dependent role in directing Th17 differentiation. In addition to studying the role of TCR signal strength in relevant Th17-mediated disease models, future studies will need to investigate how TCR signal strength regulates the expression of the Th17 master regulator of transcription, RAR-related Orphan Receptor gamma t (RORγt).

A growing number of studies indicate that TCR signal strength does not dictate T cell effector function at the individual T cell level (11, 98, 199–202). Rather, potent TCR signals are proposed to regulate the number of T cells recruited into the effector cell pool and the ratio of effector phenotypes throughout the T cell response. Therefore, TCR signal strength may regulate the overall magnitude and function of T cell responses at a population level. It is possible that antigen doses and TCR affinity promote different “selection” or “survival” strategies to shape Th cell responses at a population level. For example, high antigen dose stimulation generally seems to favor Tfh over Th1 populations *in vivo*. As Th1 cells are susceptible to AICD following high antigen dose stimulation (142, 143), the induction of BCL-6 in these conditions might confer a selection-advantage by promoting Th cell survival (203) and resistance to AICD, as has been proposed by Keck et al. (115). In support of this, BCL-6 has been reported to regulate the survival of multiple cell types (204, 205), and is known to be predictive of the long-term survival of T cells into the memory phase (191, 206, 207).

High TCR affinity generally seems to promote commitment to Th1 over Tfh and Th17 lineages. As IL-12 has long-been proposed as a polarizing agent for the selective survival and expansion of Th1 populations (208, 209), it is possible that TCR affinity regulates the ratio of Th cell effectors at the population level by controlling the receptivity to IL-12. In this way the selective expansion of high affinity Th1 populations may mask the the generation of lower affinity Th2 populations (210). Discrepancies regarding the role for TCR signal strength in CD4^+^ T cell differentiation may result from differences in how T cell responses are analyzed between different studies (at the population vs. individual cell level). Furthermore, analyses of Th cell responses at a single time-point or in a single location is likely to lead to confounding observations. Future studies need to take an unbiased approach to studying the role of TCR signal strength by tracking the T cell response across multiple time-points, tissues and at both the individual and population levels.

## Concluding Remarks

T cells must integrate distinct signals to coordinate their proliferation and differentiation. A deeper understanding of the molecular mechanisms underpinning various signaling pathways not only sheds new insights into the biology of TCR signal transduction, but also paves the way for manipulating T cell function for immunotherapy. Future studies will need to delineate the model-dependent, relative contribution of TCR signaling strength and polarizing cytokines to CD4^+^ T cell differentiation. As is the case with the regulation of the IL-12R and commitment to Th1 differentiation, van Panhuys et al. (9) has proposed that TCR signal strength may dominantly control Th cell polarization by regulating the expression of cytokine receptors important for the polarization of other Th subsets. Whilst this is an attractive hypothesis, it may only apply to the generation of some but not all Th cell subsets. For example, strong TCR signaling has been shown to both enhance and suppress the expression of the IL-4 receptor or its transcript (165, 211–213). Furthermore, it would be critical to investigate how TCR signal strength spatiotemporally regulates the T helper cell response. Different lymphoid and non-lymphoid sites vary in their cellular composition and environmental factors. Therefore, it is likely that T cell turnover and differentiation vary across different stages of infection and in different locations.

## Author Contributions

NB and CF wrote and edited this manuscript.

## Conflict of Interest

The authors declare that the research was conducted in the absence of any commercial or financial relationships that could be construed as a potential conflict of interest.

## References

[1] Zhu J Yamane H Paul WE. Differentiation of effector CD4 T cell populations. Ann Rev Immunol. (2010) 28:445. 10.1146/annurev-immunol-030409-101212 20192806 PMC3502616

[2] Corse E Gottschalk RA Allison JP. Strength of TCR-peptide/MHC interactions and *in vivo* T cell responses. J Immunol. (2011) 186:5039–45. 10.4049/jimmunol.1003650 21505216

[3] Sloan-Lancaster J Allen PM. Altered peptide ligand-induced partial T cell activation: molecular mechanisms and role in T cell biology. Annu Rev Immunol. (1996) 14:1–27. 10.1146/annurev.immunol.14.1.1 8717505

[4] van Panhuys N Klauschen F Germain RN. T-cell-receptor-dependent signal intensity dominantly controls CD4(+) T cell polarization *In vivo*. Immunity. (2014) 41:63–74. 10.1016/j.immuni.2014.06.003 24981853 PMC4114069

[5] Vigano S Utzschneider DT Perreau M Pantaleo G Zehn D Harari A. Functional avidity: a measure to predict the efficacy of effector T cells? Clin Dev Immunol. (2012) 2012:153863. 10.1155/2012/153863 23227083 PMC3511839

[6] Slifka MK Whitton JL. Functional avidity maturation of CD8(+) T cells without selection of higher affinity TCR. Nat Immunol. (2001) 2:711–7. 10.1038/90650 11477407

[7] Govern CC Paczosa MK Chakraborty AK Huseby ES. Fast on-rates allow short dwell time ligands to activate T cells. Proc Natl Acad Sci USA. (2010) 107:8724–9. 10.1073/pnas.1000966107 20421471 PMC2889346

[8] Henrickson SE Mempel TR Mazo IB Liu B Artyomov MN Zheng H. T cell sensing of antigen dose governs interactive behavior with dendritic cells and sets a threshold for T cell activation. Nat Immunol. (2008) 9:282–91. 10.1038/ni1559 18204450 PMC2698867

[9] van Panhuys N. TCR Signal Strength Alters T-DC Activation and Interaction Times and Directs the Outcome of Differentiation. Front Immunol. (2016) 7:6. 10.3389/fimmu.2016.00006 26834747 PMC4725058

[10] Sibener LV Fernandes RA Kolawole EM Carbone CB Liu F McAffee D. Isolation of a structural mechanism for uncoupling T cell receptor signaling from peptide-MHC Binding. Cell. (2018) 174:672–87 e627. 10.1016/j.cell.2018.06.017 30053426 PMC6140336

[11] Tubo NJ Pagan AJ Taylor JJ Nelson RW Linehan JL Ertelt JM. Single naive CD4+ T cells from a diverse repertoire produce different effector cell types during infection. Cell. (2013) 153:785–96. 10.1016/j.cell.2013.04.007 23663778 PMC3766899

[12] Zikherman J Au-Yeung B. The role of T cell receptor signaling thresholds in guiding T cell fate decisions. Curr Opin Immunol. (2015) 33:43–8. 10.1016/j.coi.2015.01.012 25660212 PMC4397149

[13] Rosenthal KM Edwards LJ Sabatino JJ Jr Hood JD Wasserman HA Zhu C. Low 2-dimensional CD4 T cell receptor affinity for myelin sets in motion delayed response kinetics. PLoS One. (2012) 7:e32562. 10.1371/journal.pone.0032562 22412888 PMC3296730

[14] Rosette C Werlen G Daniels MA Holman PO Alam SM Travers PJ. The impact of duration versus extent of TCR occupancy on T cell activation: a revision of the kinetic proofreading model. Immunity. (2001) 15:59–70. 10.1016/S1074-7613(01)00173-X 11485738

[15] van der Merwe PA Dushek O. Mechanisms for T cell receptor triggering. Nat Rev Immunol. (2011) 11:47–55. 10.1038/nri2887 21127503

[16] Cantrell D. Signaling in lymphocyte activation. Cold Spring Harb Perspect Biol. (2015) 7:a018788. 10.1101/cshperspect.a018788 26032717 PMC4448613

[17] Ngoenkam J Schamel WW Pongcharoen S. Selected signalling proteins recruited to the T-cell receptor-CD3 complex. Immunology. (2018) 153:42–50. 10.1111/imm.12809 28771705 PMC5721247

[18] Kim ST Shin Y Brazin K Mallis RJ Sun ZY Wagner G. TCR mechanobiology: torques and tunable structures linked to early T cell signaling. Front Immunol. (2012) 3:76. 10.3389/fimmu.2012.00076 22566957 PMC3342345

[19] Liu B Chen W Evavold BD Zhu C. Accumulation of dynamic catch bonds between TCR and agonist peptide-MHC triggers T cell signaling. Cell. (2014) 157:357–68. 10.1016/j.cell.2014.02.053 24725404 PMC4123688

[20] Wang JH Reinherz EL. The structural basis of alphabeta T-lineage immune recognition: TCR docking topologies, mechanotransduction, and co-receptor function. Immunol Rev. (2012) 250:102–19. 10.1111/j.1600-065X.2012.01161.x 23046125 PMC3694212

[21] Xu C Gagnon E Call ME Schnell JR Schwieters CD Carman CV. Regulation of T cell receptor activation by dynamic membrane binding of the CD3epsilon cytoplasmic tyrosine-based motif. Cell. (2008) 135:702–13. 10.1016/j.cell.2008.09.044 19013279 PMC2597348

[22] Davis SJ van der Merwe PA. The kinetic-segregation model: TCR triggering and beyond. Nat Immunol. (2006) 7:803–9. 10.1038/ni1369 16855606

[23] Nika K Soldani C Salek M Paster W Gray A Etzensperger R. Constitutively active Lck kinase in T cells drives antigen receptor signal transduction. Immunity. (2010) 32:766–77. 10.1016/j.immuni.2010.05.011 20541955 PMC2996607

[24] Varma R Campi G Yokosuka T Saito T Dustin ML. T cell receptor-proximal signals are sustained in peripheral microclusters and terminated in the central supramolecular activation cluster. Immunity. (2006) 25:117–27. 10.1016/j.immuni.2006.04.010 16860761 PMC1626533

[25] Rossjohn J Gras S Miles JJ Turner SJ Godfrey DI McCluskey J. T cell antigen receptor recognition of antigen-presenting molecules. Annu Rev Immunol. (2015) 33:169–200. 10.1146/annurev-immunol-032414-112334 25493333

[26] Chan AC Iwashima M Turck CW Weiss A. ZAP-70: a 70 kd protein-tyrosine kinase that associates with the TCR zeta chain. Cell. (1992) 71:649–62. 10.1016/0092-8674(92)90598-7 1423621

[27] Stanford SM Rapini N Bottini N Regulation of TCR signalling by tyrosine phosphatases: from immune homeostasis to autoimmunity. Immunology. (2012) 137:1–19. 10.1111/j.1365-2567.2012.03591.x PMC3449242 22862552

[28] Klammt C Novotna L Li DT Wolf M Blount A Zhang K. T cell receptor dwell times control the kinase activity of Zap70. Nat Immunol. (2015) 16:961–9. 10.1038/ni.3231 26237552 PMC4605427

[29] Taylor MJ Husain K Gartner ZJ Mayor S Vale RD. A DNA-based T cell receptor reveals a role for receptor clustering in ligand discrimination. Cell. (2017) 169:108–19 e120. 10.1016/j.cell.2017.03.006 28340336 PMC6934412

[30] Katz ZB Novotna L Blount A Lillemeier BF. A cycle of Zap70 kinase activation and release from the TCR amplifies and disperses antigenic stimuli. Nat Immunol. (2017) 18:86–95. 10.1038/ni.3631 27869819 PMC5490839

[31] Yokosuka T Sakata-Sogawa K Kobayashi W Hiroshima M Hashimoto-Tane A Tokunaga M. Newly generated T cell receptor microclusters initiate and sustain T cell activation by recruitment of Zap70 and SLP-76. Nat Immunol. (2005) 6:1253–62. 10.1038/ni1272 16273097

[32] Shim EK Jung SH Lee JR. Role of two adaptor molecules SLP-76 and LAT in the PI3K signaling pathway in activated T cells. J Immunol. (2011) 186:2926–35. 10.4049/jimmunol.1001785 21282515

[33] Brownlie RJ Zamoyska R. T cell receptor signalling networks: branched, diversified and bounded. Nat Rev Immunol. (2013) 13:257–69. 10.1038/nri3403 23524462

[34] Kane LP Shapiro VS Stokoe D Weiss A. Induction of NF-kappaB by the Akt/PKB kinase. Curr Biol. (1999) 9:601–4. 10.1016/S0960-9822(99)80265-6 10359702

[35] Paul S Schaefer BC. A new look at T cell receptor signaling to nuclear factor-kappaB. Trends Immunol. (2013) 34:269–81. 10.1016/j.it.2013.02.002 23474202 PMC3674144

[36] Wegener E Oeckinghaus A Papadopoulou N Lavitas L Schmidt-Supprian M Ferch U. Essential role for IkappaB kinase beta in remodeling Carma1-Bcl10-Malt1 complexes upon T cell activation. Mol Cell. (2006) 23:13–23. 10.1016/j.molcel.2006.05.027 16818229

[37] Berg LJ Finkelstein LD Lucas JA Schwartzberg PL. Tec family kinases in T lymphocyte development and function. Annu Rev Immunol. (2005) 23:549–600. 10.1146/annurev.immunol.22.012703.104743 15771581

[38] Conley JM Gallagher MP Berg LJ. T Cells and Gene Regulation: The Switching On and Turning Up of Genes after T Cell Receptor Stimulation in CD8 T Cells. Front Immunol. (2016) 7:76. 10.3389/fimmu.2016.00076 26973653 PMC4770016

[39] Aramburu J García-Cózar F Raghavan A Okamura H Rao A Hogan PG. Selective inhibition of NFAT activation by a peptide spanning the calcineurin targeting site of NFAT. Mol Cell. (1998) 1:627–37. 10.1016/S1097-2765(00)80063-5 9660947

[40] Krishna S Zhong X. Role of diacylglycerol kinases in T cell development and function. Crit Rev Immunol. (2013) 33:97–118. 10.1615/CritRevImmunol.2013006696 23582058 PMC3689416

[41] Shaulian E Karin M. AP-1 as a regulator of cell life and death. Nat Cell Biol. (2002) 4:E131–6. 10.1038/ncb0502-e131 11988758

[42] Altan-Bonnet G Germain RN. Modeling T cell antigen discrimination based on feedback control of digital ERK responses. PLoS Biol. (2005) 3:e356. 10.1371/journal.pbio.0030356 16231973 PMC1262625

[43] Coward J Germain RN Altan-Bonnet G. Perspectives for computer modeling in the study of T cell activation. Cold Spring Harb Perspect Biol. (2010) 2:a005538. 10.1101/cshperspect.a005538 20516137 PMC2869519

[44] Adams JJ Narayanan S Liu B Birnbaum ME Kruse AC Bowerman NA. T cell receptor signaling is limited by docking geometry to peptide-major histocompatibility complex. Immunity. (2011) 35:681–93. 10.1016/j.immuni.2011.09.013 22101157 PMC3253265

[45] Ma R Kellner AV Ma VP Su H Deal BR Brockman JM. DNA probes that store mechanical information reveal transient piconewton forces applied by T cells. Proc Natl Acad Sci USA. (2019) 116:16949–54. 10.1073/pnas.1904034116 31391300 PMC6708336

[46] Hong J Persaud SP Horvath S Allen PM Evavold BD Zhu C. Force-regulated *in situ* TCR-peptide-bound MHC class II kinetics determine functions of CD4+ T cells. J Immunol. (2015) 195:3557–64. 10.4049/jimmunol.1501407 26336148 PMC4592802

[47] Pullen RH III Abel SM. Catch bonds at t cell interfaces: impact of surface reorganization and membrane fluctuations. Biophys J. (2017) 113:120–31. 10.1016/j.bpj.2017.05.023 28700910 PMC5510709

[48] Glassman CR Parrish HL Lee MS Kuhns MS. Reciprocal TCR-CD3 and CD4 engagement of a nucleating pMHCII stabilizes a functional receptor macrocomplex. Cell Rep. (2018) 22:1263–75. 10.1016/j.celrep.2017.12.104 29386113 PMC5813697

[49] Chau LA Bluestone JA Madrenas J. Dissociation of intracellular signaling pathways in response to partial agonist ligands of the T cell receptor. J Exp Med. (1998) 187:1699–709. 10.1084/jem.187.10.1699 9584148 PMC2212283

[50] Hemmer B Stefanova I Vergelli M Germain RN Martin R. Relationships among TCR ligand potency, thresholds for effector function elicitation, and the quality of early signaling events in human T cells. J Immunol. (1998) 160:5807–14. 9637491

[51] Sloan-Lancaster J Shaw AS Rothbard JB Allen PM. Partial T cell signaling: altered phospho-zeta and lack of zap70 recruitment in APL-induced T cell anergy. Cell. (1994) 79:913–22. 10.1016/0092-8674(94)90080-9 8001128

[52] O'Donoghue GP Pielak RM Smoligovets AA Lin JJ Groves JT. Direct single molecule measurement of TCR triggering by agonist pMHC in living primary T cells. Elife. (2013) 2:e00778. 10.7554/eLife.00778.017 23840928 PMC3701909

[53] Stefanova I Hemmer B Vergelli M Martin R Biddison WE Germain RN. TCR ligand discrimination is enforced by competing ERK positive and SHP-1 negative feedback pathways. Nat Immunol. (2003) 4:248–54. 10.1038/ni895 12577055

[54] Plas DR Johnson R Pingel JT Matthews RJ Dalton M Roy G. Direct regulation of ZAP-70 by SHP-1 in T cell antigen receptor signaling. Science. (1996) 272:1173–6. 10.1126/science.272.5265.1173 8638162

[55] Huppa JB Gleimer M Sumen C Davis MM. Continuous T cell receptor signaling required for synapse maintenance and full effector potential. Nat Immunol. (2003) 4:749–55. 10.1038/ni951 12858171

[56] Gamper CJ Powell JD. All PI3Kinase signaling is not mTOR: dissecting mTOR-dependent and independent signaling pathways in T cells. Front Immunol. (2012) 3:312. 10.3389/fimmu.2012.00312 23087689 PMC3466461

[57] Chi H. Regulation and function of mTOR signalling in T cell fate decisions. Nat Rev Immunol. (2012) 12:325–38. 10.1038/nri3198 22517423 PMC3417069

[58] Waickman AT Powell JD. Mammalian target of rapamycin integrates diverse inputs to guide the outcome of antigen recognition in T cells. J Immunol. (2012) 188:4721–9. 10.4049/jimmunol.1103143 22556133 PMC3347776

[59] Turner MS Kane LP Morel PA. Dominant role of antigen dose in CD4+Foxp3+ regulatory T cell induction and expansion. J Immunol. (2009) 183:4895–903. 10.4049/jimmunol.0901459 19801514 PMC3142864

[60] Boomer JS Green JM. An enigmatic tail of CD28 signaling. Cold Spring Harb Perspect Biol. (2010) 2:a002436. 10.1101/cshperspect.a002436 20534709 PMC2908766

[61] Harada Y Tokushima M Matsumoto Y Ogawa S Otsuka M Hayashi K. Critical requirement for the membrane-proximal cytosolic tyrosine residue for CD28-mediated costimulation *in vivo*. J Immunol. (2001) 166:3797–803. 10.4049/jimmunol.166.6.3797 11238622

[62] Frauwirth KA Riley JL Harris MH Parry RV Rathmell JC Plas DR. The CD28 signaling pathway regulates glucose metabolism. Immunity. (2002) 16:769–77. 10.1016/S1074-7613(02)00323-0 12121659

[63] Colombetti S Basso V Mueller DL Mondino A. Prolonged TCR/CD28 engagement drives IL-2-independent T cell clonal expansion through signaling mediated by the mammalian target of rapamycin. J Immunol. (2006) 176:2730–8. 10.4049/jimmunol.176.5.2730 16493028

[64] Boise LH Minn AJ Noel PJ June CH Accavitti MA Lindsten T. CD28 costimulation can promote T cell survival by enhancing the expression of Bcl-XL. Immunity. (1995) 3:87–98. 10.1016/1074-7613(95)90161-2 7621080

[65] Hayden MS Ghosh S. NF-kappaB in immunobiology. Cell Res. (2011) 21:223–44. 10.1038/cr.2011.13 21243012 PMC3193440

[66] Marinari B Costanzo A Marzano V Piccolella E Tuosto L. CD28 delivers a unique signal leading to the selective recruitment of RelA and p52 NF-kappaB subunits on IL-8 and Bcl-xL gene promoters. Proc Natl Acad Sci USA. (2004) 101:6098–103. 10.1073/pnas.0308688101 15079071 PMC395929

[67] Wan YY DeGregori J. The survival of antigen-stimulated T cells requires NFkappaB-mediated inhibition of p73 expression. Immunity. (2003) 18:331–42. 10.1016/S1074-7613(03)00053-0 12648451

[68] Kingeter LM Paul S Maynard SK Cartwright NG Schaefer BC. Cutting edge: TCR ligation triggers digital activation of NF-kappaB. J Immunol. (2010) 185:4520–4. 10.4049/jimmunol.1001051 20855880 PMC2950878

[69] Gett AV Sallusto F Lanzavecchia A Geginat J. T cell fitness determined by signal strength. Nat Immunol. (2003) 4:355–60. 10.1038/ni908 12640450

[70] Grumont RJ Gerondakis S. Rel induces interferon regulatory factor 4 (IRF-4) expression in lymphocytes: modulation of interferon-regulated gene expression by rel/nuclear factor kappaB. J Exp Med. (2000) 191:1281–92. 10.1084/jem.191.8.1281 10770796 PMC2193138

[71] Krishnamoorthy V Kannanganat S Maienschein-Cline M Cook SL Chen J Bahroos N. The IRF4 gene regulatory module functions as a read-write integrator to dynamically coordinate T helper cell fate. Immunity. (2017) 47:481–97 e487. 10.1016/j.immuni.2017.09.001 28930660 PMC5661949

[72] Cheng J Montecalvo A Kane LP. Regulation of NF-kappaB induction by TCR/CD28. Immunol Res. (2011) 50:113–7. 10.1007/s12026-011-8216-z 21717079 PMC4066383

[73] Narayan P Holt B Tosti R Kane LP. CARMA1 is required for Akt-mediated NF-kappaB activation in T cells. Mol Cell Biol. (2006) 26:2327–36. 10.1128/MCB.26.6.2327-2336.2006 16508008 PMC1430296

[74] Cheng J Phong B Wilson DC Hirsch R Kane LP. Akt fine-tunes NF-kappaB-dependent gene expression during T cell activation. J Biol Chem. (2011) 286:36076–85. 10.1074/jbc.M111.259549 21862580 PMC3195567

[75] Dan HC Cooper MJ Cogswell PC Duncan JA Ting JP Baldwin AS. Akt-dependent regulation of NF-{kappa}B is controlled by mTOR and Raptor in association with IKK. Genes Dev. (2008) 22:1490–500. 10.1101/gad.1662308 18519641 PMC2418585

[76] Yao S Buzo BF Pham D Jiang L Taparowsky EJ Kaplan MH. Interferon regulatory factor 4 sustains CD8(+) T cell expansion and effector differentiation. Immunity. (2013) 39:833–45. 10.1016/j.immuni.2013.10.007 24211184 PMC3855863

[77] Irvine DJ Purbhoo MA Krogsgaard M Davis MM. Direct observation of ligand recognition by T cells. Nature. (2002) 419:845–9. 10.1038/nature01076 12397360

[78] Wulfing C Rabinowitz JD Beeson C Sjaastad MD McConnell HM Davis MM. Kinetics and extent of T cell activation as measured with the calcium signal. J Exp Med. (1997) 185:1815–25. 10.1084/jem.185.10.1815 9151707 PMC2196319

[79] Chen JL Morgan AJ Stewart-Jones G Shepherd D Bossi G Wooldridge L. Ca2+ release from the endoplasmic reticulum of NY-ESO-1-specific T cells is modulated by the affinity of TCR and by the use of the CD8 coreceptor. J Immunol. (2010) 184:1829–39. 10.4049/jimmunol.0902103 20053942 PMC4222200

[80] Jayaraman T Ondriasova E Ondrias K Harnick DJ Marks AR. The inositol 1,4,5-trisphosphate receptor is essential for T-cell receptor signaling. Proc Natl Acad Sci USA. (1995) 92:6007–11. 10.1073/pnas.92.13.6007 7597070 PMC41631

[81] Brogdon JL Leitenberg D Bottomly K. The potency of TCR signaling differentially regulates NFATc/p activity and early IL-4 transcription in naive CD4+ T cells. J Immunol. (2002) 168:3825–32. 10.4049/jimmunol.168.8.3825 11937535

[82] Nurieva RI Chuvpilo S Wieder ED Elkon KB Locksley R Serfling E. A costimulation-initiated signaling pathway regulates NFATc1 transcription in T lymphocytes. J Immunol. (2007) 179:1096–103. 10.4049/jimmunol.179.2.1096 17617602

[83] Fiering S Northrop JP Nolan GP Mattila PS Crabtree GR Herzenberg LA. Single cell assay of a transcription factor reveals a threshold in transcription activated by signals emanating from the T-cell antigen receptor. Genes Dev. (1990) 4:1823–34. 10.1101/gad.4.10.1823 2123468

[84] Fuhrmann F Lischke T Gross F Scheel T Bauer L Kalim KW. Adequate immune response ensured by binary IL-2 and graded CD25 expression in a murine transfer model. Elife. (2016) 5:e20616. 10.7554/eLife.20616.017 28035902 PMC5201416

[85] James JR. Tuning ITAM multiplicity on T cell receptors can control potency and selectivity to ligand density. Sci Signal. (2018) 11:aan1088. 10.1126/scisignal.aan1088 29789296 PMC6517276

[86] Pielak RM O'Donoghue GP Lin JJ Alfieri KN Fay NC Low-Nam ST. Early T cell receptor signals globally modulate ligand:receptor affinities during antigen discrimination. Proc Natl Acad Sci USA. (2017) 114:12190–5. 10.1073/pnas.1613140114 29087297 PMC5699024

[87] Podtschaske M Benary U Zwinger S Hofer T Radbruch A Baumgrass R. Digital NFATc2 activation per cell transforms graded T cell receptor activation into an all-or-none IL-2 expression. PLoS ONE. (2007) 2:e935. 10.1371/journal.pone.0000935 17895976 PMC1978524

[88] Salazar C Hofer T. Allosteric regulation of the transcription factor NFAT1 by multiple phosphorylation sites: a mathematical analysis. J Mol Biol. (2003) 327:31–45. 10.1016/S0022-2836(03)00085-8 12614606

[89] Das J Ho M Zikherman J Govern C Yang M Weiss A. Digital signaling and hysteresis characterize ras activation in lymphoid cells. Cell. (2009) 136:337–51. 10.1016/j.cell.2008.11.051 19167334 PMC2662698

[90] Tkach KE Barik D Voisinne G Malandro N Hathorn MM Cotari JW. T cells translate individual, quantal activation into collective, analog cytokine responses via time-integrated feedbacks. Elife. (2014) 3:e01944. 10.7554/eLife.01944.018 24719192 PMC3980879

[91] Chuvpilo S Jankevics E Tyrsin D Akimzhanov A Moroz D Jha MK. Autoregulation of NFATc1/A expression facilitates effector T cells to escape from rapid apoptosis. Immunity. (2002) 16:881–95. 10.1016/S1074-7613(02)00329-1 12121669

[92] Zhou B Cron RQ Wu B Genin A Wang Z Liu S. Regulation of the murine Nfatc1 gene by NFATc2. J Biol Chem. (2002) 277:10704–11. 10.1074/jbc.M107068200 11786533

[93] Kim HP Leonard WJ. The basis for TCR-mediated regulation of the IL-2 receptor alpha chain gene: role of widely separated regulatory elements. EMBO J. (2002) 21:3051–9. 10.1093/emboj/cdf321 12065418 PMC126074

[94] Schuh K Twardzik T Kneitz B Heyer J Schimpl A Serfling E. The interleukin 2 receptor alpha chain/CD25 promoter is a target for nuclear factor of activated T cells. J Exp Med. (1998) 188:1369–73. 10.1084/jem.188.7.1369 9763616 PMC2212486

[95] Castellanos MC Munoz C Montoya MC Lara-Pezzi E Lopez-Cabrera M de Landazuri MO. Expression of the leukocyte early activation antigen CD69 is regulated by the transcription factor AP-1. J Immunol. (1997) 159:5463–73. 9580241

[96] Allison KA Sajti E Collier JG Gosselin D Troutman TD Stone EL. Affinity and dose of TCR engagement yield proportional enhancer and gene activity in CD4+ T cells. Elife. (2016) 5:e10134. 10.7554/eLife.10134 27376549 PMC4931909

[97] Marangoni F Murooka TT Manzo T Kim EY Carrizosa E Elpek NM. The transcription factor NFAT exhibits signal memory during serial T cell interactions with antigen-presenting cells. Immunity. (2013) 38:237–49. 10.1016/j.immuni.2012.09.012 23313588 PMC3582823

[98] Huang J Brameshuber M Zeng X Xie J Li QJ Chien YH. A single peptide-major histocompatibility complex ligand triggers digital cytokine secretion in CD4(+) T cells. Immunity. (2013) 39:846–57. 10.1016/j.immuni.2013.08.036 24120362 PMC3846396

[99] Guy CS Vignali KM Temirov J Bettini ML Overacre AE Smeltzer M. Distinct TCR signaling pathways drive proliferation and cytokine production in T cells. Nat Immunol. (2013) 14:262–70. 10.1038/ni.2538 23377202 PMC3577985

[100] Demotz S Grey HM Sette A. The minimal number of class II MHC-antigen complexes needed for T cell activation. Science. (1990) 249:1028–30. 10.1126/science.2118680 2118680

[101] Harding CV Unanue ER. Quantitation of antigen-presenting cell MHC class II/peptide complexes necessary for T-cell stimulation. Nature. (1990) 346:574–6. 10.1038/346574a0 2115981

[102] Mayya V Dustin ML. What Scales the T Cell Response? Trends Immunol. (2016) 37:513–22. 10.1016/j.it.2016.06.005 27364960

[103] Ozga AJ Moalli F Abe J Swoger J Sharpe J Zehn D. pMHC affinity controls duration of CD8+ T cell-DC interactions and imprints timing of effector differentiation versus expansion. J Exp Med. (2016) 213:2811–29. 10.1084/jem.20160206 27799622 PMC5110015

[104] Zehn D Lee SY Bevan MJ. Complete but curtailed T-cell response to very low-affinity antigen. Nature. (2009) 458:211–4. 10.1038/nature07657 19182777 PMC2735344

[105] King CG Koehli S Hausmann B Schmaler M Zehn D Palmer E. T cell affinity regulates asymmetric division, effector cell differentiation, and tissue pathology. Immunity. (2012) 37:709–20. 10.1016/j.immuni.2012.06.021 23084359 PMC3622938

[106] Baumgartner CK Yagita H Malherbe LP. A TCR affinity threshold regulates memory CD4 T cell differentiation following vaccination. J Immunol. (2012) 189:2309–17. 10.4049/jimmunol.1200453 22844120 PMC3424363

[107] Fazilleau N McHeyzer-Williams LJ Rosen H McHeyzer-Williams MG. The function of follicular helper T cells is regulated by the strength of T cell antigen receptor binding. Nat Immunol. (2009) 10:375–84. 10.1038/ni.1704 19252493 PMC2712297

[108] Kotov DI Mitchell JS Pengo T Ruedl C Way SS Langlois RA. TCR affinity biases th cell differentiation by regulating CD25, Eef1e1, and Gbp2. J Immunol. (2019) 202:2535–45. 10.4049/jimmunol.1801609 30858199 PMC6478541

[109] Malherbe L Hausl C Teyton L McHeyzer-Williams MG. Clonal selection of helper T cells is determined by an affinity threshold with no further skewing of TCR binding properties. Immunity. (2004) 21:669–79. 10.1016/j.immuni.2004.09.008 15539153

[110] Malherbe L Mark L Fazilleau N McHeyzer-Williams LJ McHeyzer-Williams MG. Vaccine adjuvants alter TCR-based selection thresholds. Immunity. (2008) 28:698–709. 10.1016/j.immuni.2008.03.014 18450485 PMC2695494

[111] Ploquin MJ Eksmond U Kassiotis G. B cells and TCR avidity determine distinct functions of CD4+ T cells in retroviral infection. J Immunol. (2011) 187:3321–30. 10.4049/jimmunol.1101006 21841129 PMC3173872

[112] McHeyzer-Williams LJ Panus JF Mikszta JA McHeyzer-Williams MG. Evolution of antigen-specific T cell receptors *in vivo*: preimmune and antigen-driven selection of preferred complementarity-determining region 3 (CDR3) motifs. J Exp Med. (1999) 189:1823–38. 10.1084/jem.189.11.1823 10359586 PMC2193074

[113] Fassò M Anandasabapathy N Crawford F Kappler J Fathman CG Ridgway WM. T cell receptor (TCR)-mediated repertoire selection and loss of TCR vβ diversity during the initiation of a CD4+ T cell response *in vivo*. J Exp Med. (2000) 192:1719–30. 10.1084/jem.192.12.1719 11120769 PMC2213496

[114] Corse E Gottschalk RA Krogsgaard M Allison JP. Attenuated T cell responses to a high-potency ligand *in vivo*. PLoS Biol. (2010) 8. 10.1371/journal.pbio.1000481 20856903 PMC2939023

[115] Keck S Schmaler M Ganter S Wyss L Oberle S Huseby ES. Antigen affinity and antigen dose exert distinct influences on CD4 T-cell differentiation. Proc Natl Acad Sci USA. (2014) 111:14852–7. 10.1073/pnas.1403271111 25267612 PMC4205596

[116] Quiel J Caucheteux S Laurence A Singh NJ Bocharov G Ben-Sasson SZ. Antigen-stimulated CD4 T-cell expansion is inversely and log-linearly related to precursor number. Proc Natl Acad Sci U S A. (2011) 108:3312–7. 10.1073/pnas.1018525108 21292989 PMC3044359

[117] Rees W Bender J Teague TK Kedl RM Crawford F Marrack P. An inverse relationship between T cell receptor affinity and antigen dose during CD4(+) T cell responses *in vivo* and *in vitro*. Proc Natl Acad Sci U S A. (1999) 96:9781–6. 10.1073/pnas.96.17.9781 10449771 PMC22287

[118] Skokos D Shakhar G Varma R Waite JC Cameron TO Lindquist RL. Peptide-MHC potency governs dynamic interactions between T cells and dendritic cells in lymph nodes. Nat Immunol. (2007) 8:835–44. 10.1038/ni1490 17632517

[119] Heinzel S Marchingo JM Horton MB Hodgkin PD. The regulation of lymphocyte activation and proliferation. Curr Opin Immunol. (2018) 51:32–8. 10.1016/j.coi.2018.01.002 29414529

[120] Bonnevier JL Mueller DL. Cutting edge: B7/CD28 interactions regulate cell cycle progression independent of the strength of TCR signaling. J Immunol. (2002) 169:6659–63. 10.4049/jimmunol.169.12.6659 12471093

[121] Deenick EK Gett AV Hodgkin PD. Stochastic model of T cell proliferation: a calculus revealing IL-2 regulation of precursor frequencies, cell cycle time, and survival. J Immunol. (2003) 170:4963–72. 10.4049/jimmunol.170.10.4963 12734339

[122] Iezzi G Karjalainen K Lanzavecchia A. The duration of antigenic stimulation determines the fate of naive and effector T cells. Immunity. (1998) 8:89–95. 10.1016/S1074-7613(00)80461-6 9462514

[123] Hommel M Hodgkin PD. TCR affinity promotes CD8+ T cell expansion by regulating survival. J Immunol. (2007) 179:2250–60. 10.4049/jimmunol.179.4.2250 17675486

[124] Gett AV Hodgkin PD. A cellular calculus for signal integration by T cells. Nat Immunol. (2000) 1:239–44. 10.1038/79782 10973282

[125] Marchingo JM Kan A Sutherland RM Duffy KR Wellard CJ Belz GT. T cell signaling. Antigen affinity, costimulation, and cytokine inputs sum linearly to amplify T cell expansion. Science. (2014) 346:1123–7. 10.1126/science.1260044 25430770

[126] Mishima T Fukaya S Toda S Ando Y Matsunaga T Inobe M. Rapid G0/1 transition and cell cycle progression in CD8(+) T cells compared to CD4(+) T cells following *in vitro* stimulation. Microbiol Immunol. (2017) 61:168–75. 10.1111/1348-0421.12479 28370382

[127] Appleman LJ van Puijenbroek AA Shu KM Nadler LM Boussiotis VA. CD28 costimulation mediates down-regulation of p27kip1 and cell cycle progression by activation of the PI3K/PKB signaling pathway in primary human T cells. J Immunol. (2002) 168:2729–36. 10.4049/jimmunol.168.6.2729 11884439

[128] Hashemolhosseini S Nagamine Y Morley SJ Desrivieres S Mercep L Ferrari S. Rapamycin inhibition of the G1 to S transition is mediated by effects on cyclin D1 mRNA and protein stability. J Biol Chem. (1998) 273:14424–9. 10.1074/jbc.273.23.14424 9603954

[129] Shi M Lin TH Appell KC Berg LJ. Cell cycle progression following naive T cell activation is independent of Jak3/common gamma-chain cytokine signals. J Immunol. (2009) 183:4493–501. 10.4049/jimmunol.0804339 19734221 PMC2768578

[130] Yang K Shrestha S Zeng H Karmaus PW Neale G Vogel P. T cell exit from quiescence and differentiation into Th2 cells depend on Raptor-mTORC1-mediated metabolic reprogramming. Immunity. (2013) 39:1043–56. 10.1016/j.immuni.2013.09.015 24315998 PMC3986063

[131] Colombetti S Benigni F Basso V Mondino A. Clonal anergy is maintained independently of T cell proliferation. J Immunol. (2002) 169:6178–86. 10.4049/jimmunol.169.11.6178 12444122

[132] Zheng Y Collins SL Lutz MA Allen AN Kole TP Zarek PE. A role for mammalian target of rapamycin in regulating T cell activation versus anergy. J Immunol. (2007) 178:2163–70. 10.4049/jimmunol.178.4.2163 17277121

[133] Rowell EA Wells AD. The role of cyclin-dependent kinases in T-cell development, proliferation, and function. Crit Rev Immunol. (2006) 26:189–212. 10.1615/CritRevImmunol.v26.i3.10 16928186

[134] Heinzel S Binh Giang T Kan A Marchingo JM Lye BK Corcoran LM. A Myc-dependent division timer complements a cell-death timer to regulate T cell and B cell responses. Nat Immunol. (2017) 18:96–103. 10.1038/ni.3598 27820810

[135] Preston GC Sinclair LV Kaskar A Hukelmann JL Navarro MN Ferrero I. Single cell tuning of Myc expression by antigen receptor signal strength and interleukin-2 in T lymphocytes. EMBO J. (2015) 34:2008–24. 10.15252/embj.201490252 26136212 PMC4551349

[136] Grumont R Lock P Mollinari M Shannon FM Moore A Gerondakis S. The mitogen-induced increase in T cell size involves PKC and NFAT activation of Rel/NF-kappaB-dependent c-myc expression. Immunity. (2004) 21:19–30. 10.1016/j.immuni.2004.06.004 15345217

[137] Wang R Dillon CP Shi LZ Milasta S Carter R Finkelstein D. The transcription factor Myc controls metabolic reprogramming upon T lymphocyte activation. Immunity. (2011) 35:871–82. 10.1016/j.immuni.2011.09.021 22195744 PMC3248798

[138] Iwata A Durai V Tussiwand R Briseno CG Wu X Grajales-Reyes GE. Quality of TCR signaling determined by differential affinities of enhancers for the composite BATF-IRF4 transcription factor complex. Nat Immunol. (2017) 18:563–72. 10.1038/ni.3714 28346410 PMC5401770

[139] Wu H Witzl A Ueno H. Assessment of TCR signal strength of antigen-specific memory CD8(+) T cells in human blood. Blood Adv. (2019) 3:2153–63. 10.1182/bloodadvances.2019000292 31320320 PMC6650739

[140] Man K Miasari M Shi W Xin A Henstridge DC Preston S. The transcription factor IRF4 is essential for TCR affinity-mediated metabolic programming and clonal expansion of T cells. Nat Immunol. (2013) 14:1155–65. 10.1038/ni.2710 24056747

[141] Han S Asoyan A Rabenstein H Nakano N Obst R. Role of antigen persistence and dose for CD4+ T-cell exhaustion and recovery. Proc Natl Acad Sci USA. (2010) 107:20453–8. 10.1073/pnas.1008437107 21059929 PMC2996637

[142] Zhang X Brunner T Carter L Dutton RW Rogers P Bradley L. Unequal death in T helper cell (Th) 1 and Th2 effectors: Th1, but not Th2, effectors undergo rapid Fas/FasL-mediated apoptosis. J Exp Med. (1997) 185:1837–49. 10.1084/jem.185.10.1837 9151709 PMC2196321

[143] Refaeli Y Van Parijs L Alexander SI Abbas AK. Interferon gamma is required for activation-induced death of T lymphocytes. J Exp Med. (2002) 196:999–1005. 10.1084/jem.20020666 12370261 PMC2194022

[144] Gallegos AM Xiong H Leiner IM Susac B Glickman MS Pamer EG. Control of T cell antigen reactivity via programmed TCR downregulation. Nat Immunol. (2016) 17:379–86. 10.1038/ni.3386 26901151 PMC4803589

[145] Kedl RM. T cells compete for access to antigen-bearing antigen-presenting cells. J Exp Med. (2000) 192:1105–13. 10.1084/jem.192.8.1105 11034600 PMC2195874

[146] Thorborn G Ploquin MJ Eksmond U Pike R Bayer W Dittmer U. Clonotypic composition of the CD4+ T cell response to a vectored retroviral antigen is determined by its speed. J Immun. (2014) 193:1567–77. 10.4049/jimmunol.1400667 25000983 PMC4119786

[147] Andargachew R Martinez RJ Kolawole EM Evavold BD. CD4 T Cell Affinity Diversity Is Equally Maintained during Acute and Chronic Infection. J Immunol. (2018) 201:19–30. 10.4049/jimmunol.1800295 29777029 PMC6497530

[148] Martinez RJ Andargachew R Martinez HA Evavold BD. Low-affinity CD4+ T cells are major responders in the primary immune response. Nat Commun. (2016) 7:13848. 10.1038/ncomms13848 27976744 PMC5234832

[149] Martinez RJ Evavold BD. Lower Affinity T Cells are Critical Components and Active Participants of the Immune Response. Front Immunol. (2015) 6:468. 10.3389/fimmu.2015.00468 26441973 PMC4564719

[150] Sabatino JJ Jr Huang J Zhu C Evavold BD. High prevalence of low affinity peptide-MHC II tetramer-negative effectors during polyclonal CD4+ T cell responses. J Exp Med. (2011) 208:81–90. 10.1084/jem.20101574 21220453 PMC3023139

[151] Ertelt JM Johanns TM Mysz MA Nanton MR Rowe JH Aguilera MN. Selective culling of high avidity antigen-specific CD4+ T cells after virulent Salmonella infection. Immunology. (2011) 134:487–97. 10.1111/j.1365-2567.2011.03510.x 22044420 PMC3230801

[152] Merkenschlager J Ploquin MJ Eksmond U Andargachew R Thorborn G Filby A. Stepwise B-cell-dependent expansion of T helper clonotypes diversifies the T-cell response. Nat Commun. (2016) 7:10281. 10.1038/ncomms10281 26728651 PMC4728444

[153] Gottschalk RA Corse E Allison JP. TCR ligand density and affinity determine peripheral induction of Foxp3 *in vivo*. J Exp Med. (2010) 207:1701–11. 10.1084/jem.20091999 20660617 PMC2916126

[154] Gottschalk RA Hathorn MM Beuneu H Corse E Dustin ML Altan-Bonnet G. Distinct influences of peptide-MHC quality and quantity on *in vivo* T-cell responses. Proc Natl Acad Sci USA. (2012) 109:881–6. 10.1073/pnas.1119763109 22223661 PMC3271915

[155] Constant S Pfeiffer C Woodard A Pasqualini T Bottomly K. Extent of T cell receptor ligation can determine the functional differentiation of naive CD4+ T cells. J Exp Med. (1995) 182:1591–6. 10.1084/jem.182.5.1591 7595230 PMC2192213

[156] Ise W Totsuka M Sogawa Y Ametani A Hachimura S Sato T. Naive CD4+ T cells exhibit distinct expression patterns of cytokines and cell surface molecules on their primary responses to varying doses of antigen. J Immunol. (2002) 168:3242–50. 10.4049/jimmunol.168.7.3242 11907078

[157] Ruedl C Bachmann MF Kopf M. The antigen dose determines T helper subset development by regulation of CD40 ligand. Eur J Immunol. (2000) 30:2056–64. 10.1002/1521-4141(200007)30:7<2056::AID-IMMU2056>3.0.CO2-S 10940895

[158] Tao X Grant C Constant S Bottomly K. Induction of IL-4-producing CD4+ T cells by antigenic peptides altered for TCR binding. J Immunol. (1997) 158:4237–44. 9126985

[159] Yamane H Zhu J Paul WE. Independent roles for IL-2 and GATA-3 in stimulating naive CD4+ T cells to generate a Th2-inducing cytokine environment. J Exp Med. (2005) 202:793–804. 10.1084/jem.20051304 16172258 PMC2212937

[160] Rogers PR Huston G Swain SL. High antigen density and IL-2 are required for generation of CD4 effectors secreting Th1 rather than Th0 cytokines. J Immun. (1998) 161:3844–52. 9780149

[161] Hosken NA Shibuya K Heath AW Murphy KM O'Garra A. The effect of antigen dose on CD4+ T helper cell phenotype development in a T cell receptor-alpha beta-transgenic model. J Exp Med. (1995) 182:1579–84. 10.1084/jem.182.5.1579 7595228 PMC2192218

[162] Rogers PR Croft M. Peptide dose, affinity, and time of differentiation can contribute to the Th1/Th2 cytokine balance. J Immunol. (1999) 163:1205–13. 10415015

[163] Yamashita M Kimura M Kubo M Shimizu C Tada T Perlmutter RM. T cell antigen receptor-mediated activation of the Ras/mitogen-activated protein kinase pathway controls interleukin 4 receptor function and type-2 helper T cell differentiation. Proc Natl Acad Sci USA. (1999) 96:1024–9. 10.1073/pnas.96.3.1024 9927687 PMC15344

[164] Yamashita M Hashimoto K Kimura M Kubo M Tada T Nakayama T. Requirement for p56 (lck) tyrosine kinase activation in Th subset differentiation. Int Immunol. (1998) 10:577–91. 10.1093/intimm/10.5.577 9645606

[165] Yamashita M Katsumata M Iwashima M Kimura M Shimizu C Kamata T. T cell receptor-induced calcineurin activation regulates T helper type 2 cell development by modifying the interleukin 4 receptor signaling complex. J Exp Med. (2000) 191:1869–79. 10.1084/jem.191.11.1869 10839803 PMC2213529

[166] Nakayama T Yamashita M. The TCR-mediated signaling pathways that control the direction of helper T cell differentiation. Semin Immunol. (2010) 22:303–9. 10.1016/j.smim.2010.04.010 20488727

[167] Jorritsma PJ Brogdon JL Bottomly K. Role of TCR-induced extracellular signal-regulated kinase activation in the regulation of early IL-4 expression in naive CD4+ T cells. The Journal of Immunology. (2003) 170:2427–34. 10.4049/jimmunol.170.5.2427 12594266

[168] Kumar V Bhardwaj V Soares L Alexander J Sette A Sercarz E. Major histocompatibility complex binding affinity of an antigenic determinant is crucial for the differential secretion of interleukin 4/5 or interferon gamma by T cells. Proc Natl Acad Sci U S A. (1995) 92:9510–4. 10.1073/pnas.92.21.9510 7568164 PMC40831

[169] Leitenberg D Boutin Y Constant S Bottomly K. CD4 regulation of TCR signaling and T cell differentiation following stimulation with peptides of different affinities for the TCR. J Immunol. (1998) 161:1194–203. 9686579

[170] Tao X Constant S Jorritsma P Bottomly K. Strength of TCR signal determines the costimulatory requirements for Th1 and Th2 CD4+ T cell differentiation. J Immun. (1997) 159:5956–63. 9550393

[171] Pfeiffer C Stein J Southwood S Ketelaar H Sette A Bottomly K. Altered peptide ligands can control CD4 T lymphocyte differentiation *in vivo*. J Exp Med. (1995) 181:1569–74. 10.1084/jem.181.4.1569 7699337 PMC2191965

[172] Blander JM Sant'Angelo DB Bottomly K Janeway CA Jr. Alteration at a single amino acid residue in the T cell receptor alpha chain complementarity determining region 2 changes the differentiation of naive CD4 T cells in response to antigen from T helper cell type 1 (Th1) to Th2. J Exp Med. (2000) 191:2065–74. 10.1084/jem.191.12.2065 10859331 PMC2193209

[173] Ahlers JD Belyakov IM Matsui S Berzofsky JA. Signals delivered through TCR instruct IL-12 receptor (IL-12R) expression: IL-12 and tumor necrosis factor-alpha synergize for IL-12R expression at low antigen dose. Int Immunol. (2001) 13:1433–42. 10.1093/intimm/13.11.1433 11675375

[174] Ahlers JD Belyakov IM Thomas EK Berzofsky JA. High-affinity T helper epitope induces complementary helper and APC polarization, increased CTL, and protection against viral infection. J Clin Invest. (2001) 108:1677–85. 10.1172/JCI200113463 11733563 PMC200990

[175] Fujimoto N Ishida H Nakamura I Ogasawara K Itoh Y. Quantities of interleukin-12p40 in mature CD8alpha negative dendritic cells correlate with strength of TCR signal and determine Th cell development. Microbiol Immunol. (2003) 47:1017–24. 10.1111/j.1348-0421.2003.tb03462.x 14695452

[176] Hwang ES Szabo SJ Schwartzberg PL Glimcher LH. T helper cell fate specified by kinase-mediated interaction of T-bet with GATA-3. Science. (2005) 307:430–3. 10.1126/science.1103336 15662016

[177] Kanhere A Hertweck A Bhatia U Gokmen MR Perucha E Jackson I. T-bet and GATA3 orchestrate Th1 and Th2 differentiation through lineage-specific targeting of distal regulatory elements. Nat Commun. (2012) 3:1268. 10.1038/ncomms2260 23232398 PMC3535338

[178] Usui T Preiss JC Kanno Y Yao ZJ Bream JH O'Shea JJ. T-bet regulates Th1 responses through essential effects on GATA-3 function rather than on IFNG gene acetylation and transcription. J Exp Med. (2006) 203:755–66. 10.1084/jem.20052165 16520391 PMC2118252

[179] Ismail N Bretscher PA. The Th1/Th2 nature of concurrent immune responses to unrelated antigens can be independent. J Immunol. (1999) 163:4842–50. 10528185

[180] Lagrange PH Mackaness GB Miller TE. Influence of dose and route of antigen injection on the immunological induction of T cells. J Exp Med. (1974) 139:528–42. 10.1084/jem.139.3.528 4591170 PMC2139541

[181] Menon JN Bretscher PA. Parasite dose determines the Th1/Th2 nature of the response to Leishmania major independently of infection route and strain of host or parasite. Eur J Immunol. (1998) 28:4020–28. 9862338 10.1002/(SICI)1521-4141(199812)28:12<4020::AID-IMMU4020>3.0.CO;2-3

[182] Parish CR Liew FY. Immune response to chemically modified flagellin. 3. Enhanced cell-mediated immunity during high and low zone antibody tolerance to flagellin. J Exp Med. (1972) 135:298–311. 10.1084/jem.135.2.298 5060292 PMC2180527

[183] Crotty S. Follicular helper CD4 T cells (TFH). Annu Rev Immunol. (2011) 29:621–63. 10.1146/annurev-immunol-031210-101400 21314428

[184] King IL Mohrs M. IL-4-producing CD4+ T cells in reactive lymph nodes during helminth infection are T follicular helper cells. J Exp Med. (2009) 206:1001–7. 10.1084/jem.20090313 19380638 PMC2715031

[185] Reinhardt RL Liang HE Locksley RM. Cytokine-secreting follicular T cells shape the antibody repertoire. Nat Immunol. (2009) 10:385–93. 10.1038/ni.1715 19252490 PMC2714053

[186] Baumjohann D Preite S Reboldi A Ronchi F Ansel KM Lanzavecchia A. Persistent antigen and germinal center B cells sustain T follicular helper cell responses and phenotype. Immunity. (2013) 38:596–605. 10.1016/j.immuni.2012.11.020 23499493

[187] Benson RA MacLeod MK Hale BG Patakas A Garside P Brewer JM. Antigen presentation kinetics control T cell/dendritic cell interactions and follicular helper T cell generation *in vivo*. Elife. (2015) 4:e06994. 10.7554/eLife.06994.015 26258879 PMC4558563

[188] DiToro D Winstead CJ Pham D Witte S Andargachew R Singer JR. Differential IL-2 expression defines developmental fates of follicular versus nonfollicular helper T cells. Science. (2018) 361:eaao2933. 10.1126/science.aao2933 30213884 PMC6501592

[189] Johnston RJ Choi YS Diamond JA Yang JA Crotty S. STAT5 is a potent negative regulator of TFH cell differentiation. J Exp Med. (2012) 209:243–50. 10.1084/jem.20111174 22271576 PMC3281266

[190] Nagaoka M Hatta Y Kawaoka Y Malherbe LP. Antigen signal strength during priming determines effector CD4 T cell function and antigen sensitivity during influenza virus challenge. J Immunol. (2014) 193:2812–20. 10.4049/jimmunol.1401358 25086170 PMC4157108

[191] Pepper M Pagan AJ Igyarto BZ Taylor JJ Jenkins MK. Opposing signals from the Bcl6 transcription factor and the interleukin-2 receptor generate T helper 1 central and effector memory cells. Immunity. (2011) 35:583–95. 10.1016/j.immuni.2011.09.009 22018468 PMC3208313

[192] Snook JP Kim C Williams MA. TCR signal strength controls the differentiation of CD4(+) effector and memory T cells. Sci Immunol. (2018) 3:eaas9103. 10.1126/sciimmunol.aas9103 30030369 PMC6126666

[193] Iezzi G Sonderegger I Ampenberger F Schmitz N Marsland BJ Kopf M. CD40-CD40L cross-talk integrates strong antigenic signals and microbial stimuli to induce development of IL-17-producing CD4+ T cells. Proc Natl Acad Sci USA. (2009) 106:876–81. 10.1073/pnas.0810769106 19136631 PMC2630101

[194] Kastirr I Crosti M Maglie S Paroni M Steckel B Moro M. Signal Strength and Metabolic requirements control cytokine-induced Th17 differentiation of uncommitted human T cells. J Immunol. (2015) 195:3617–27. 10.4049/jimmunol.1501016 26378072

[195] Gomez-Rodriguez J Wohlfert EA Handon R Meylan F Wu JZ Anderson SM. Itk-mediated integration of T cell receptor and cytokine signaling regulates the balance between Th17 and regulatory T cells. J Exp Med. (2014) 211:529–43. 10.1084/jem.20131459 24534190 PMC3949578

[196] Purvis HA Stoop JN Mann J Woods S Kozijn AE Hambleton S. Low-strength T-cell activation promotes Th17 responses. Blood. (2010) 116:4829–37. 10.1182/blood-2010-03-272153 20713963 PMC3223073

[197] Revu S Wu J Henkel M Rittenhouse N Menk A Delgoffe GM. IL-23 and IL-1beta drive human Th17 cell differentiation and metabolic reprogramming in absence of CD28 COstimulation. Cell Rep. (2018) 22:2642–53. 10.1016/j.celrep.2018.02.044 29514093 PMC5884137

[198] Tibbitt C Falconer J Stoop J van Eden W Robinson JH Hilkens CM. Reduced TCR-dependent activation through citrullination of a T-cell epitope enhances Th17 development by disruption of the STAT3/5 balance. Eur J Immunol. (2016) 46:1633–43. 10.1002/eji.201546217 27173727 PMC4949576

[199] Cho YL Flossdorf M Kretschmer L Hofer T Busch DH Buchholz VR. TCR signal quality modulates fate decisions of single CD4(+) T cells in a probabilistic manner. Cell Rep. (2017) 20:806–18. 10.1016/j.celrep.2017.07.005 28746867

[200] Gerlach C Rohr JC Perie L van Rooij N van Heijst JW Velds A. Heterogeneous differentiation patterns of individual CD8+ T cells. Science. (2013) 340:635–9. 10.1126/science.1235487 23493421

[201] Richard AC Lun ATL Lau WWY Gottgens B Marioni JC Griffiths GM. T cell cytolytic capacity is independent of initial stimulation strength. Nat Immunol. (2018) 19:849–58. 10.1038/s41590-018-0160-9 30013148 PMC6300116

[202] Prlic M Hernandez-Hoyos G Bevan MJ. Duration of the initial TCR stimulus controls the magnitude but not functionality of the CD8+ T cell response. J Exp Med. (2006) 203:2135–43. 10.1084/jem.20060928 16908626 PMC2118397

[203] Hollister K Kusam S Wu H Clegg N Mondal A Sawant DV. Insights into the role of Bcl6 in follicular Th cells using a new conditional mutant mouse model. J Immunol. (2013) 191:3705–11. 10.4049/jimmunol.1300378 23980208 PMC3783642

[204] Albagli-Curiel O. Ambivalent role of BCL6 in cell survival and transformation. Oncogene. (2003) 22:507–16. 10.1038/sj.onc.1206152 12555064

[205] Zhu B Zhang R Li C Jiang L Xiang M Ye Z. BCL6 modulates tissue neutrophil survival and exacerbates pulmonary inflammation following influenza virus infection. Proc Natl Acad Sci USA. (2019) 116:11888–93. 10.1073/pnas.1902310116 31138703 PMC6575592

[206] Choi YS Yang JA Yusuf I Johnston RJ Greenbaum J Peters B. Bcl6 expressing follicular helper CD4 T cells are fate committed early and have the capacity to form memory. J Immunol. (2013) 190:4014–26. 10.4049/jimmunol.1202963 23487426 PMC3626566

[207] Weber JP Fuhrmann F Hutloff A. T-follicular helper cells survive as long-term memory cells. Eur J Immunol. (2012) 42:1981–8. 10.1002/eji.201242540 22730020

[208] Mullen AC High FA Hutchins AS Lee HW Villarino AV Livingston DM. Role of T-bet in Commitment of TH1 Cells Before IL-12-Dependent Selection. Science. (2001) 292:1907–10. 10.1126/science.1059835 11397944

[209] Boyton RJ Altmann DM. Is selection for TCR affinity a factor in cytokine polarization? Trends Immunol. (2002) 23:526–9. 10.1016/s1471-4906(02)02319-0 12401404

[210] Milner JD Fazilleau N McHeyzer-Williams M Paul W. Cutting edge: lack of high affinity competition for peptide in polyclonal CD4+ responses unmasks IL-4 production. J Immun. (2010) 184:6569–73. 10.4049/jimmunol.1000674 20495070 PMC2930602

[211] Gonnord P Angermann BR Sadtler K Gombos E Chappert P Meier-Schellersheim M. A hierarchy of affinities between cytokine receptors and the common gamma chain leads to pathway cross-talk. Sci Signal. (2018) 11:eaal1253. 10.1126/scisignal.aal1253 29615515

[212] Nakamura T Kamogawa Y Bottomly K Flavell RA. Polarization of IL-4- and IFN-gamma-producing CD4+ T cells following activation of naive CD4+ T cells. J Immunol. (1997) 158:1085–94. 9013946

[213] Perona-Wright G Mohrs K Mayer KD Mohrs M. Differential regulation of IL-4Ralpha expression by antigen versus cytokine stimulation characterizes Th2 progression *in vivo*. J Immunol. (2010) 184:615–23. 10.4049/jimmunol.0902408 20018622 PMC3066071

